# Supplementary material for: Prior physical synchrony enhances rapport and inter-brain synchronization during subsequent educational communication
Source: Sci Rep. 2019 Sep 4;9:12747. doi: 10.1038/s41598-019-49257-z (PMC6726616; doi:10.1038/s41598-019-49257-z)
Supplement: Supplementary file 1 — Supplementary Information [file 41598_2019_49257_MOESM1_ESM.pdf]

Supplementary Information

Manuscript title:
Prior physical synchrony enhances rapport and inter-brain synchronization during subsequent educational communication

Authors:
Takayuki Nozawa[a,b*], Kohei Sakaki[c], Shigeyuki Ikeda[b], Hyeonjeong Jeong[d], Shohei Yamazaki[e], Kelssy Hitomi dos Santos Kawata[e], Natasha Yuriko dos Santos Kawata[c], Yukako Sasaki[c], Kay Kulason[c], Kanan Hirano[e], Yoshihiro Miyake[f], Ryuta Kawashima[b,c]

Affiliations:
[a]*Research Institute for the Earth Inclusive Sensing, Tokyo Institute of Technology, Tokyo, Japan*
[b]*Department of Ubiquitous Sensing, Institute of Development, Aging and Cancer, Tohoku University, Sendai, Japan*
[c]*Department of Advanced Brain Science, Institute of Development, Aging and Cancer, Tohoku University, Sendai, Japan*
[d]*Graduate School of International Cultural Studies, Tohoku University, Sendai, Japan*
[e]*Department of Human Brain Science, Institute of Development, Aging and Cancer, Tohoku University, Sendai, Japan*
[f]*Department of Computer Science, Tokyo Institute of Technology, Tokyo, Japan*

***Corresponding author:**
Takayuki Nozawa
Research Institute for the Earth Inclusive Sensing, Tokyo Institute of Technology
2-12-1 Ookayama, Meguro-ku, Tokyo 152-8550, Japan
Tel: +81-3-5734-3048
E-mail: nozawa.t.ac@m.titech.ac.jp

Supplementary Tables

Table S1. Assignment of physical synchronous/asynchronous conditions and the pairs of tempi for the rhythmic movement block of each session for each participant pair.

Pair No.	Session 1		Session 2	
	Condition	Tempo pair (BPM)	Condition	Tempo pair (BPM)
1	Sync	(23, 23)	Async	(26, 29)
2	Async	(29, 26)	Sync	(23, 23)
3	Sync	(26, 26)	Async	(23, 29)
4	Async	(29, 23)	Sync	(26, 26)
5	Async	(26, 29)	Sync	(23, 23)
6	Sync	(29, 29)	Async	(23, 26)
7	Sync	(23, 23)	Async	(29, 26)
8	Async	(26, 23)	Sync	(29, 29)
9	Async	(23, 29)	Sync	(26, 26)
10	Sync	(26, 26)	Async	(29, 23)
11	Sync	(23, 23)	Async	(26, 29)
12	Async	(23, 26)	Sync	(29, 29)
13	Async	(29, 26)	Sync	(23, 23)
14	Sync	(29, 29)	Async	(26, 23)
15	Sync	(26, 26)	Async	(23, 29)
16	Async	(29, 23)	Sync	(26, 26)
17	Async	(26, 29)	Sync	(23, 23)
18	Sync	(29, 29)	Async	(23, 26)
19	Sync	(23, 23)	Async	(29, 26)
20	Async	(26, 23)	Sync	(29, 29)
21	Async	(23, 29)	Sync	(26, 26)
22	Sync	(26, 26)	Async	(29, 23)
23	Async	(23, 26)	Sync	(29, 29)
24	Sync	(23, 23)	Async	(26, 29)
25	Sync	(29, 29)	Async	(26, 23)
26	Async	(29, 26)	Sync	(23, 23)
27	Sync	(26, 26)	Async	(23, 29)
28	Async	(29, 23)	Sync	(26, 26)
29	Sync	(23, 23)	Async	(29, 26)
30	Async	(26, 23)	Sync	(29, 29)
31	Async	(23, 29)	Sync	(26, 26)
32	Sync	(29, 29)	Async	(26, 23)

Table S2. Words and example sentences used in the word teaching-learning blocks of the two sessions.

For Session 1

Word	Example sentence
abrogate	The country may abrogate the international agreement.
abstain	It is often difficult to abstain from drinking.
acrid	The room was filled with the acrid smell of tobacco.
adroit	He is adroit in making excuses.
ameliorate	Judge ordered the company to ameliorate working conditions.
appease	Only a sincere apology will appease my anger.
astute	The boss made an astute business decision.
austere	The church was austere and simple.

For Session 2

Word	Example sentence
cajole	The salesman will cajole you into buying the car.
callow	He is a young callow man with no experience.
candid	He is trusted because he always gives candid opinions.
capitulate	He capitulated to his wife's demand.
caustic	The teacher was known by his caustic comments.
censure	The food factory was censured for its carelessness.
choleric	He is friendly at one moment, choleric the next.
cringe	Large noise of thunder made the children cringe.

Supplementary Figures

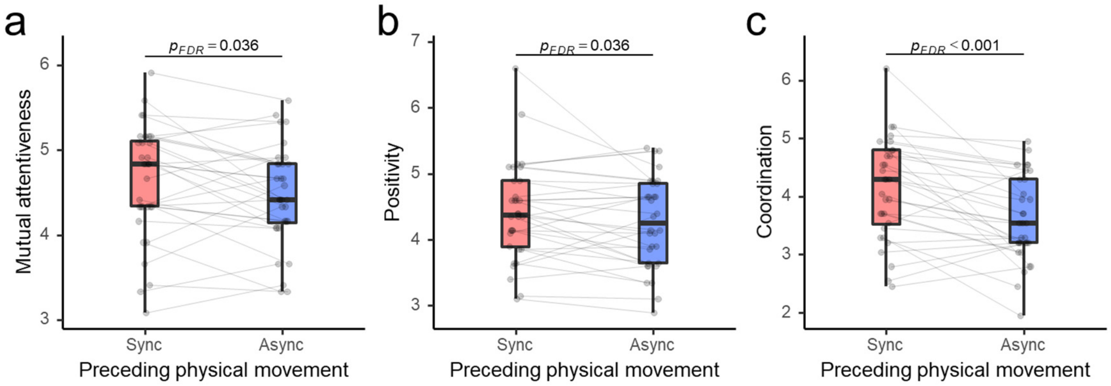

Figure S1. Three components of rapport, (a) mutual attentiveness, (b) positivity, and (c) coordination, during the word teaching-learning task after experiencing physical synchrony/asynchrony.

Box plots show the median, interquartile range (IQR), and minimum/maximum values of the average rapport ratings during the teaching-learning block of all dyads for the two conditions of the preceding rhythmic movement block. Grey points with connecting lines represent the two conditions of each dyad.

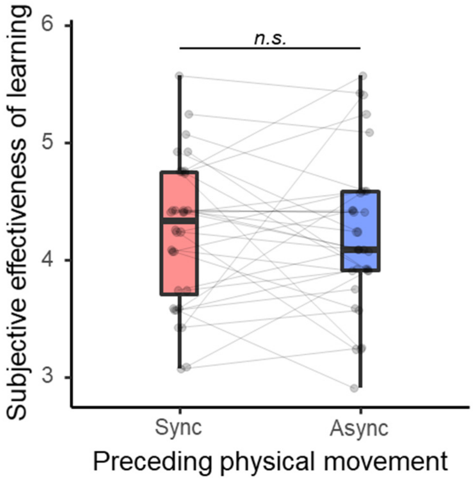

Figure S2. Subjective effectiveness of learning after experiencing physical synchrony/asynchrony.

See the caption of Fig. S1 for box plot notations.

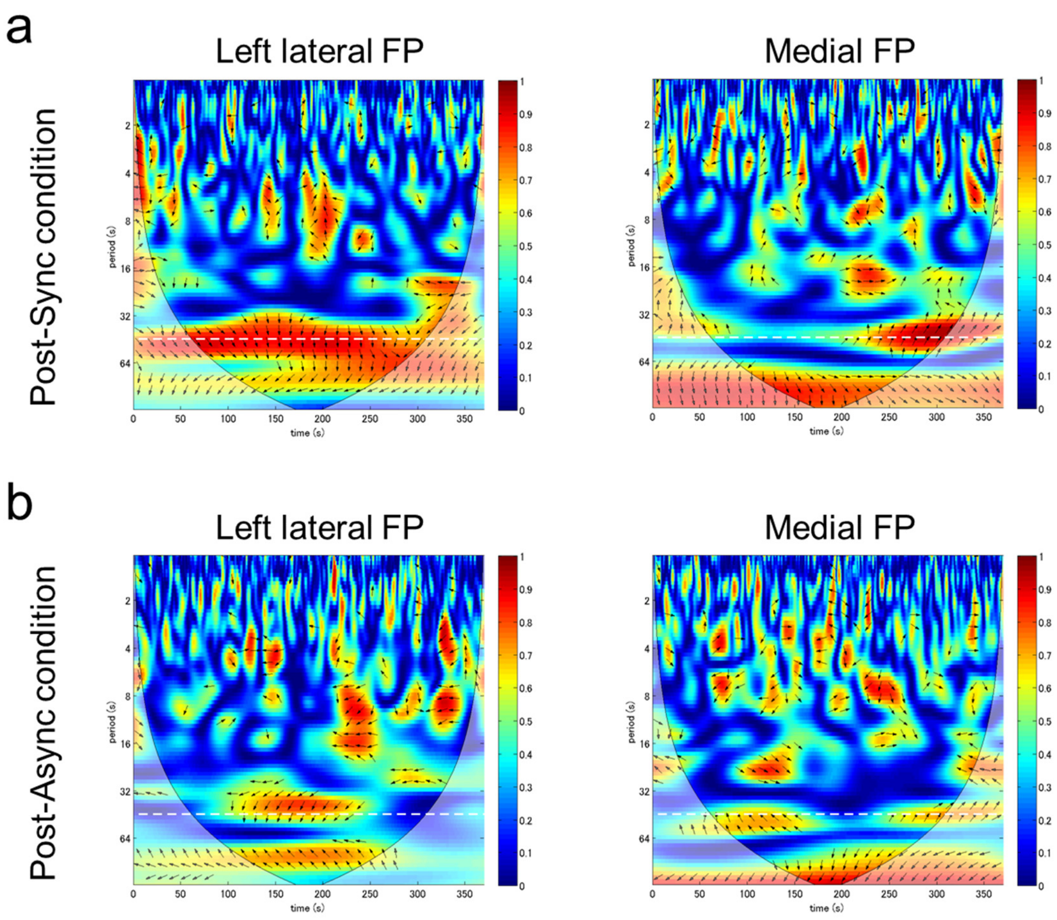

Figure S3. Illustrative examples of wavelet transform coherence (WTC) of neural signals from a single pair of participants.

Left and right panels show interpersonal coherence of the left lateral and medial PFC, respectively, during the word teaching-learning task after experiencing physical synchrony (a) and asynchrony (b). In each panel, the horizontal white dashed line at period = 45 seconds indicates the teaching-learning task period, at which WTC values were analysed. The faded colour areas bordered by spindle-like curves indicate the cones of influence (COIs), where the values can suffer from edge effects and thus were excluded from the analysis (see main text). In the areas with high coherence values, arrows indicate the phase angle of the paired signals, with right and left directions corresponding to in-phase (0) and anti-phase (π), respectively.

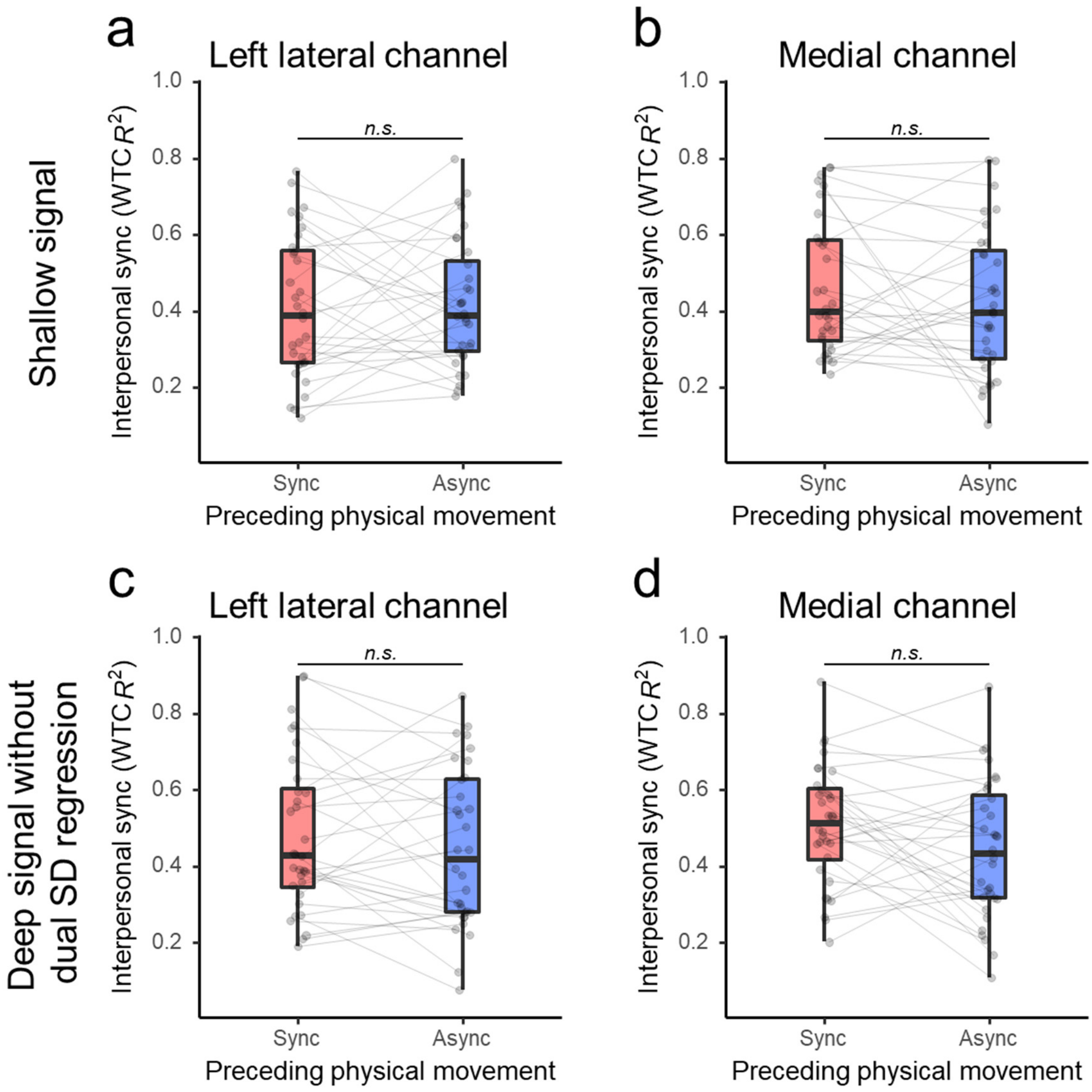

Figure S4. Interpersonal synchronization values of contaminating and contaminated signals.

Values were calculated for the shallow signals from the left lateral (a) and medial (b) 1-cm-SD channels, and for the deep signals from the left lateral (c) and medial (d) 3-cm-SD channels without the removal of shallow signal components by dual SD regression, during the word teaching-learning task after experiencing physical synchrony/asynchrony. See the caption of Fig. S1 for box plot notations.

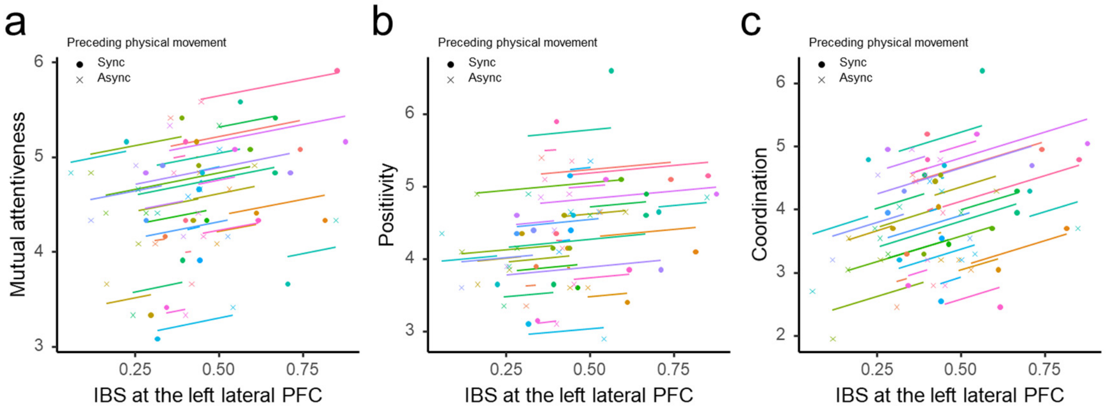

Figure S5. Within-pair correlations between IBS at the left lateral prefrontal cortex (PFC) and the ratings on the components of rapport modulated by prior synchronous/asynchronous physical movements.

Three components of rapport: (a) mutual attentiveness, (b) positivity, and (c) coordination. Coloured points (•, ×) represent the two conditions of each dyad. Coloured lines represents the best linear fit for each dyad, estimated by the repeated measures correlation, using the same slope and varying intercepts[1]

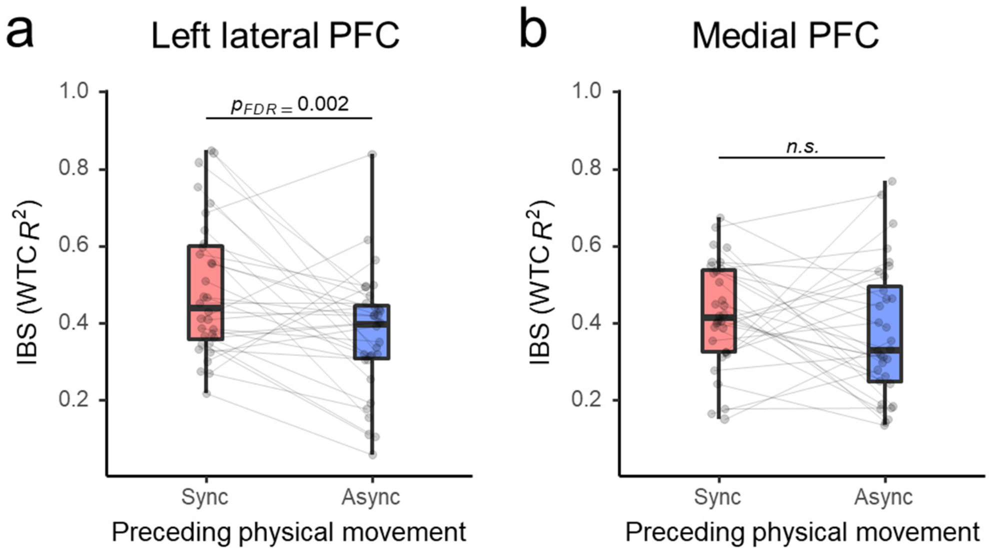

Figure S6. Post-Sync vs. post-Async inter-brain synchronization (IBS) in the left lateral prefrontal cortex (PFC; a) and the medial PFC (b), estimated using the extended period range of [40, 50] seconds.

See the caption of Fig. S1 for box plot notations. WTC, wavelet transform coherence.

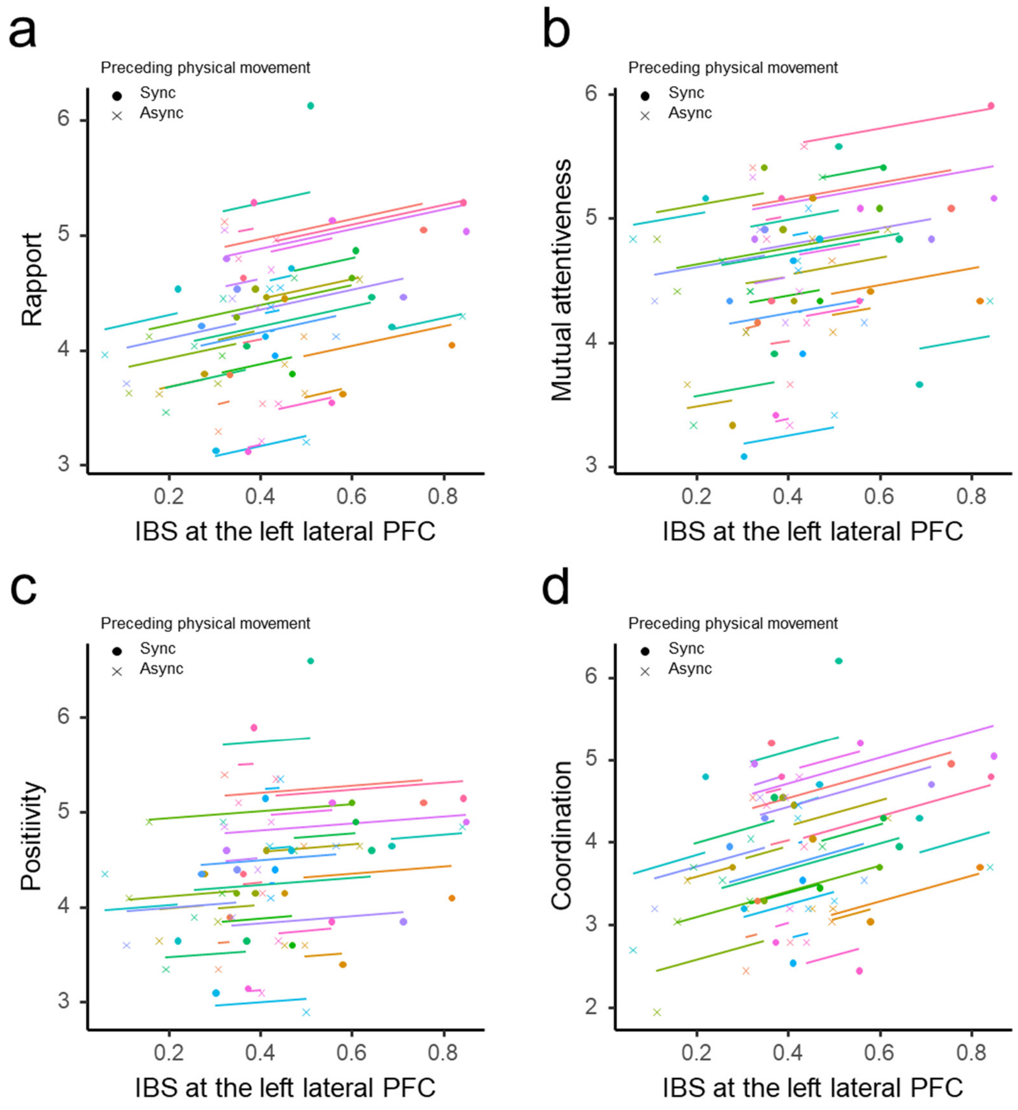

Figure S7. Within-pair correlations between IBS at the left lateral prefrontal cortex (PFC) with the extended period range of [40, 50] seconds and the ratings on rapport and its components modulated by prior synchronous/asynchronous physical movements.

Rapport (a) and its three components: (b) mutual attentiveness, (c) positivity, and (d) coordination. Coloured points (•, ×) represent the two conditions of each dyad. Coloured lines represents the best linear fit for each dyad, estimated by the repeated measures correlation, using the same slope and varying intercepts[1].

Supplementary Text

Temporal independence of IBS from shallow signal synchronization

To further confirm the neural origin of the observed IBS, we investigated whether the temporal changes in IBS were similar to the interpersonal synchronization of the shallow signals, which is expected to reflect synchronization in the physiological (e.g. arousal) and/or physical (head motion and postural) changes[2]. Temporal correlations between the interpersonal WTC time courses of brain signals obtained by using dual-SD regression[3] and the interpersonal WTC time courses of shallow signals from the 1-cm-SD pairs, at period 45 seconds, were calculated at each channel (left lateral and medial). The correlations were averaged over the two sessions, Fisher z-transformed, and subjected to a one sample t-test. There were no significant correlations for the left lateral and medial channels (left lateral: mean $r = -0.05$, $t(31) = -0.62$, $p = 0.539$; medial: mean $r = 0.07$, $t(31) = 0.82$, p=0.419).

The same correlation analysis between the interpersonal WTC time courses of deep signals obtained from the 3-cm-SD pairs without using the dual-SD regression and the interpersonal WTC time courses of shallow signals showed significant correlations (left

lateral: mean $r = 0.40$, $t(31) = 6.60$, $p = 2.2 \times 10^{-7}$; medial: mean $r = 0.43$, $t(31) = 5.38$, $p = 7.2 \times 10^{-6}$).

These results indicate that the IBS was independent from the artefactual interpersonal synchronization in the shallow tissues, while the IBS obtained without artefact removal using the dual SD regression was moderately contaminated by the artefactual synchronization in the shallow tissues.

IBS analyses with wider period of interest

To query how tightly the observed effects on IBS were coupled to the task period of 45 seconds, we conducted the same analyses as those described in the main text, but using the IBS estimates obtained by averaging WTC R^2 values over the widened period range of [40, 50] seconds.

Paired t-tests showed significantly higher IBS in the post-Sync than post-Async condition in the left lateral PFC ($t(31) = 3.30$, $p_{FDR} = 0.002$, $d_z = 0.58$, $d_{av} = 0.70$; Fig. S6a), but not in the medial PFC ($t(31) = 1.30$, $p_{FDR} > 0.05$, $d_z = 0.23$, $d_{av} = 0.30$; Fig. S6b). The results were consistent with those obtained using the WTC

estimates at the task period of 45 seconds (Fig. 4a, b), with the effect size being slightly decreased.

The repeated measures correlation analyses to test the hypothesised positive relationship between the IBS in the left lateral PFC and the degree of rapport revealed a significant positive correlation with rapport (repeated measures $r_{rm}(31) = 0.38$, $p_{FDR} = 0.018$; Fig. S7a). As for the three components of rapport, coordination showed a significant positive within-subject correlation with IBS, mutual attentiveness showed a marginal correlation, and positivity did not show a significant correlation (Mutual attentiveness: $r_{rm}(31) = 0.32$, $p_{FDR} = 0.053$; positivity $r_{rm}(31) = 0.18$, $p_{FDR} > 0.05$; coordination $r_{rm}(31) = 0.40$, $p_{FDR} = 0.034$; Fig. S7b–d). Again, the results were mostly consistent with those obtained using the WTC estimates at the task period of 45 seconds (Fig. 5 and Fig. S5a-c), but the effect size was decreased.

These results suggest that prior physical synchrony enhanced IBS focally in the task-driven neural component that is coupled tightly to the task period of 45 seconds, rather than the spontaneous neural activities which fluctuate and would thus be distributed more widely across frequencies[4].

References

1 Bakdash, J. Z. & Marusich, L. R. Repeated measures correlation. *Front Psychol* **8**, 456 (2017).

2 Nozawa, T., Sasaki, Y., Sakaki, K., Yokoyama, R. & Kawashima, R. Interpersonal frontopolar neural synchronization in group communication: An exploration toward fNIRS hyperscanning of natural interactions. *Neuroimage* **133**, 484–497 (2016).

3 Saager, R. B., Telleri, N. L. & Berger, A. J. Two-detector corrected near infrared spectroscopy (C-NIRS) detects hemodynamic activation responses more robustly than single-detector NIRS. *Neuroimage* **55**, 1679–1685 (2011).

4 Deco, G., Jirsa, V. K. & McIntosh, A. R. Emerging concepts for the dynamical organization of resting-state activity in the brain. *Nat Rev Neurosci* **12**, 43–56 (2011).
